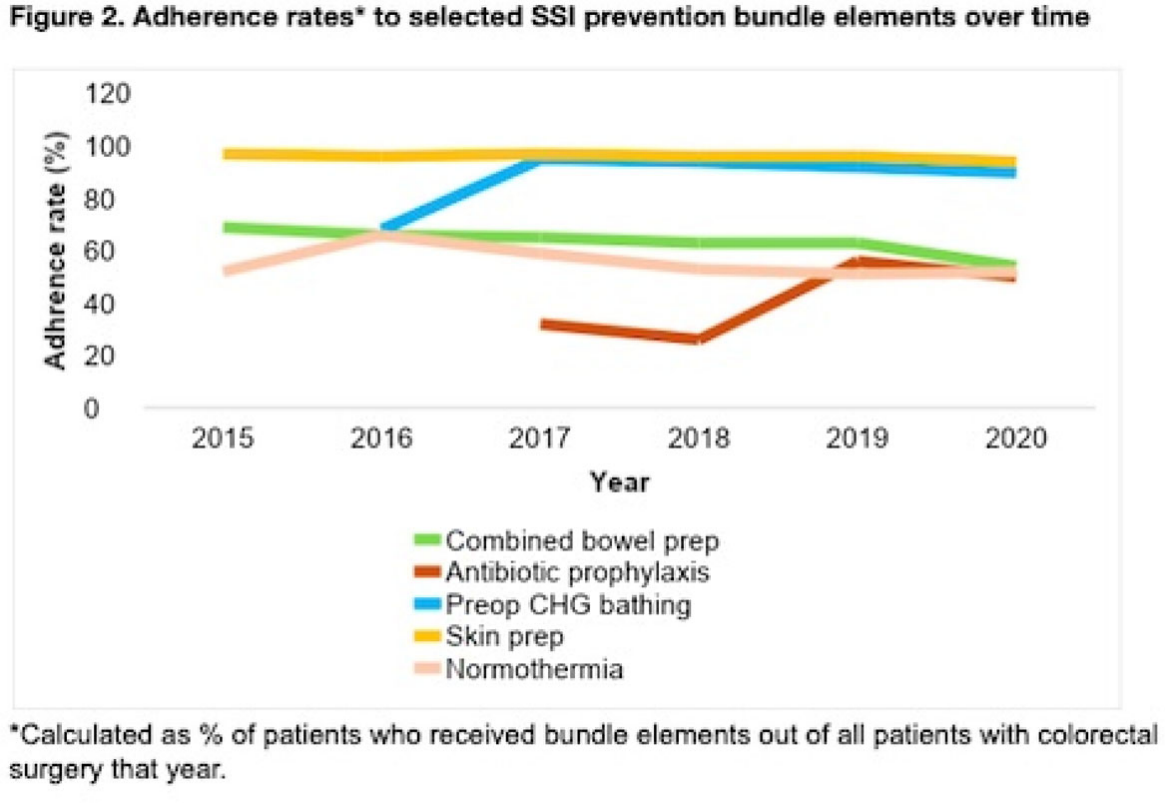# The Strike Team as an implementation strategy for surgical infection prevention

**DOI:** 10.1017/ash.2022.77

**Published:** 2022-05-16

**Authors:** Buddhi Hatharaliyadda, Michelle Schmitz, Fauzia Osman, Kenneth Van Dyke, Nasia Safdar, Aurora Pop-Vicas, Charles Heise, Anne Mork

## Abstract

**Background:** Surgical site infections (SSIs) incur up to $10 billion annually due to their excessive morbidity. SSI prevention bundles have had variable success in colorectal surgery. For example, at the University of Wisconsin Hospital, a 505-bed regional referral center, SSI rates have remained high despite the introduction of a 14-element SSI prevention bundle in 2016. To aid in the implementation of this complex bundle, the hospital started Strike Teams in 2019. We have described the impact of Strike Teams on colorectal SSI rates in our tertiary-care hospital. **Methods:** A Strike Team with key stakeholders from colorectal surgery (ie, surgeon, OR director, nurses, surgical technicians), anesthesia, pharmacy, infection prevention, and infectious disease was formed, supported by the hospital’s executive leadership. The Strike Team met monthly throughout 2019 to review each SSI case, discussed barriers to adherence for the SSI prevention bundle elements with implementation difficulties (Table 1), and proposed actionable feedback to increase adherence. The latter was disseminated to frontline clinicians by the teams’ surgical leaders during everyday clinical practice. The Strike Team was paused in 2020 due to resource reallocation in response to the COVID-19 pandemic. Monthly and quarterly SSI surveillance was conducted according to CDC guidance. **Results:** Colorectal SSI rates before, after, and during Strike Team activity are shown in Fig. 1. Adherence rates to the bundle elements targeted by the Strike Team are shown in Fig. 2. **Conclusions:** Adherence to the preferred antibiotic prophylaxis increased, although adherence to other bundle elements of focus did not change significantly. SSI rates decreased below our expectation while the Strike Team was active in our hospital, although SSI reduction was not sustained. Further research should study the effectiveness of Strike Teams as a long-term implementation strategy for SSI prevention in colorectal surgery.

**Funding:** None

**Disclosures:** None

**Table:**
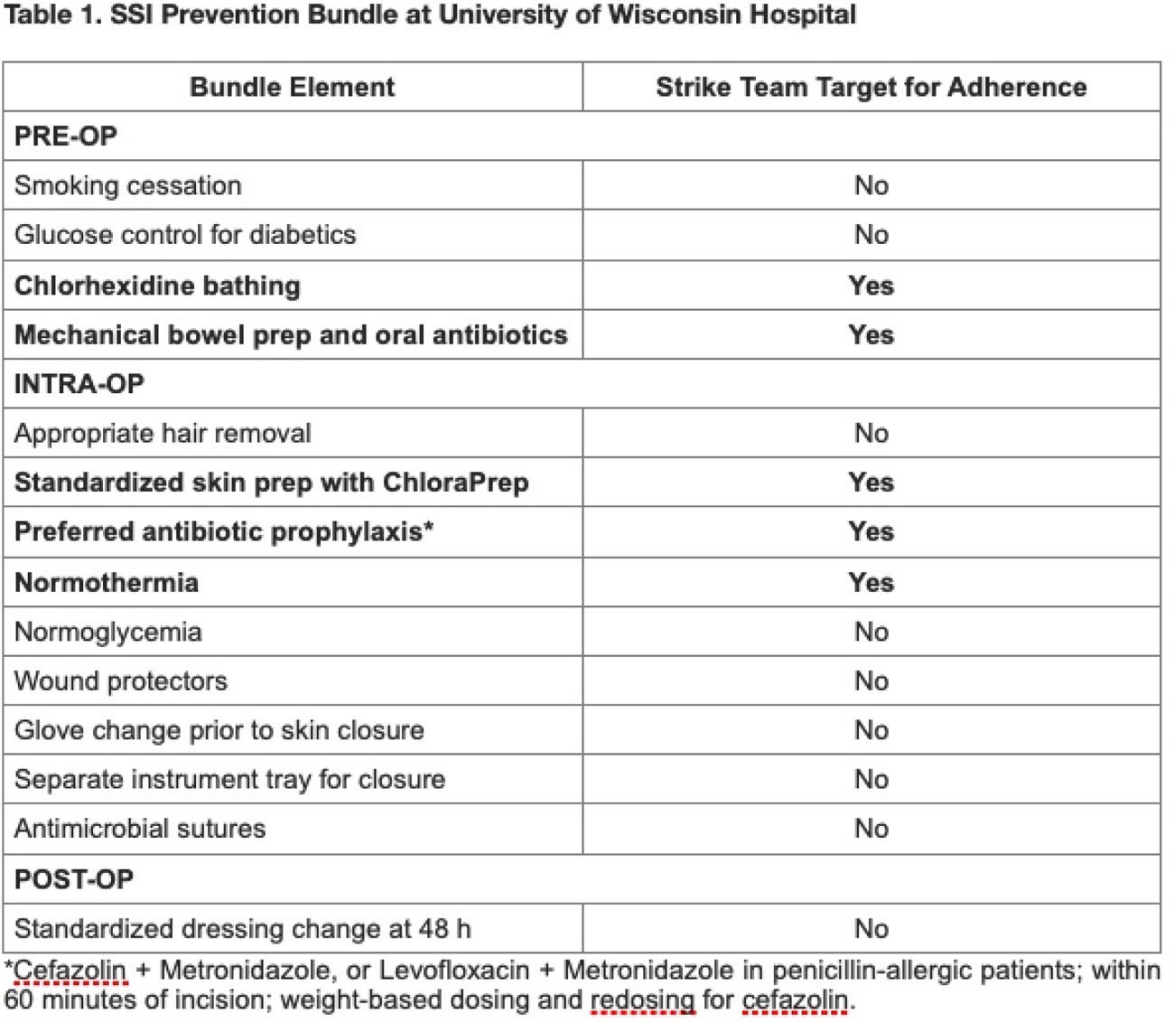

**Figure:**
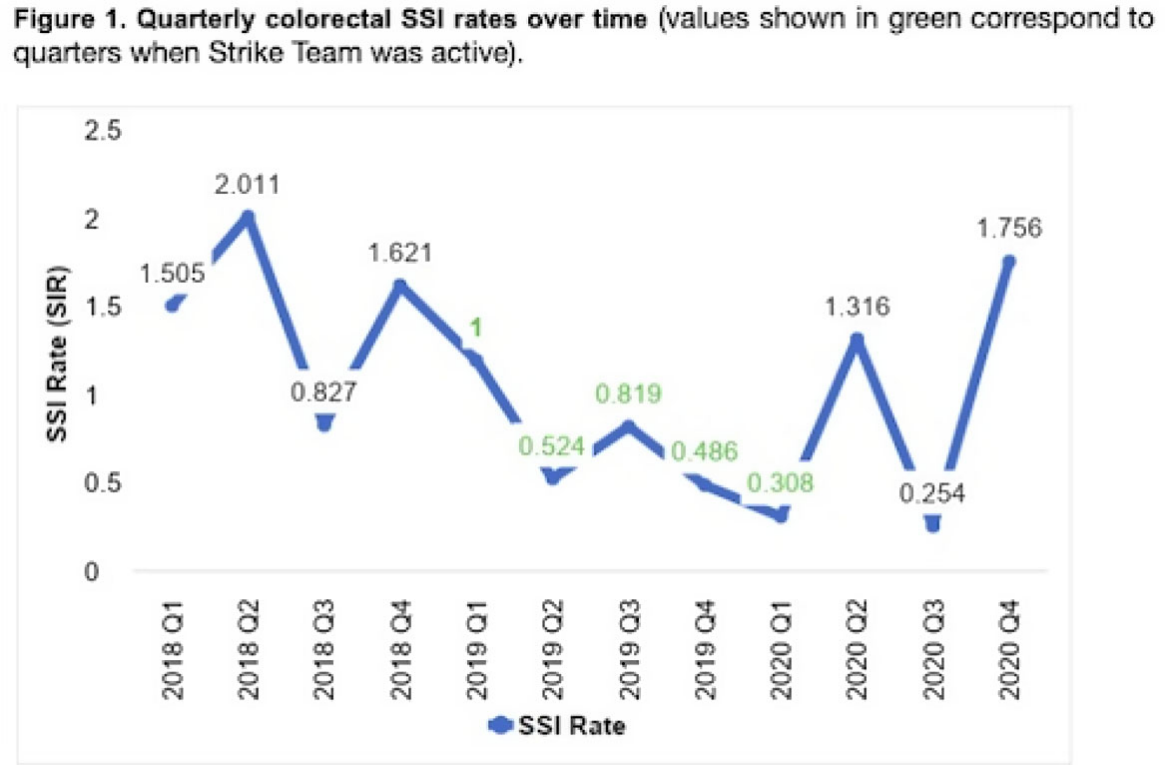

**Figure:**